# The Surgeon's Vade Mecum

**Published:** 1841-10

**Authors:** 


ART. VIII.—*The Surgeon's Vade Mecum.* By ROBERT DRUITT. Second Edition. *With Fifty Wood Engravings.—London,* 1841. 8vo, pp. 524.

ON a former occasion (Br. and For. Med. Rev., vol. VIII., 536,) we gave a favorable judgment of the first edition of this work, which the voice of the profession, in so soon demanding a second, seems to have ratified. The work is much enlarged and improved; and we can safely recommend it to the notice of the junior members of the profession, as one that may be relied on for the accuracy and fulness of its practical details.

